# Assessing Endoscopic Suture Performance of Gynecology and Obstetrics Residents Following Methodic Training

**DOI:** 10.1055/s-0043-1772476

**Published:** 2023-11-09

**Authors:** Lucas Ribeiro Nogueira, Kathiane Augusto Lustosa, Larissa Almeida Oliveira Galindo, Stephany Ellen de Castro, Liz Rodrigues Picanço, Lucas Lima da Silva, Samuel Soares Coutinho, Leonardo Robson Pinheiro Sobreira Bezerra

**Affiliations:** 1 Universidade Federal do Ceará, Departamento de Saúde Materno Infantil, Fortaleza, CE, Brazil; 2 Universidade Federal do Cariri, Faculdade de Medicina, Juazeiro do Norte, CE, Brazil

**Keywords:** suture techniques, medical education, in-service training, simulation training, laparoscopy, técnicas de sutura, educação médica, capacitação em serviço, treinamento por simulação, laparoscopia

## Abstract

**Objective** To evaluate the performance of residents in gynecology and obstetrics before and after practicing laparoscopic sutures, to establish when the training shows the best results, in addition to comparing whether being in different years of residency influences this progression.

**Methods** A prospective cohort study involving 32 medical residents evaluated with a pretest to establish their previous knowledge in laparoscopic suture. This test consisted of knotting two wires, one made of polypropylene and the other of polyglactin, with a blocking sequence of five semi-knots. We set a 30-minute limit to complete the task. Then, the residents held four training meetings, focusing on suture, Gladiator rule, knot, and symmetries, in addition to executing blocking sequences. A second test to establish progress was performed.

**Results** Regarding the time spent to make the stiches using polyglactin wire, a statistically significant time improvement ( *p* < 0.01) was observed, with a 10.67-minute pretraining median (mean 12.24 minutes) and a 2.53-minute posttraining median (mean 3.25 minutes). Regarding the stitches with polypropylene wire, a statistically significant time improvement ( *p* < 0.05) was also observed, with a 9.38-minute pretraining median (mean 15.43 minutes) and a 3.65-minute posttraining median (mean 4.54 minutes). A total of 64.2% of the residents had been able to make the knot with polypropylene previously. One hundred percent were able to complete the task in the posttest.

**Conclusion** Model training using the Gladiator rule for laparoscopic suture improves the knotting time with statistically similar performance, regardless of the year of residency, after systematic training.

## Resumo

**Objetivo** Avaliar a performance de residentes em ginecologia e obstetrícia antes e depois de praticarem suturas laparoscópicas, com o intuito de estabelecer quando o treinamento mostra os melhores resultados, comparando se estar em diferentes da residência influencia essa progressão.

**Métodos** Um estudo coorte prospectivo envolvendo 32 médicos residentes avaliados com um teste pré-treinamento para avaliar seus conhecimentos prévios em sutura laparoscópica. Esse teste consistia em atar nós em dois fios, um de polipropileno e o outro de poliglactina, com uma sequencia de bloqueio de cinco seminós. Definiu-se um limite de 30 minutos para se completar a tarefa. Depois, os residentes tiveram quatro reuniões de treinamento, focadas em sutura, técnica da Regra do Gladiador, nós e simetria, executando, ainda, uma sequência de pontos. Um segundo teste foi feito para avaliar o progresso.

**Resultados** Com relação ao tempo para realizarem os pontos com fio de poliglactina, uma melhora de tempo estatisticamente significativa ( *p* < 0.01) foi observada, com uma mediana de 10.67 minutos no pré-treinamento (média de 12.24 minutos) e uma mediana de 2.53 minutos no pós-treinamento (média de 3.25 minutos). Com relação ao fio de polipropileno, uma melhora de tempo estatisticamente significativa ( *p* < 0.05) também foi observada, com uma mediana de pré-treinamento de 9.38 minutos (média de 15.43 minutos) e uma mediana de pós-treinamento de 3.65 minutos (média de 4.54 minutos). Um total de 64.2% dos residentes foram capazes de realizar os nós com polipropileno inicialmente. Cem por cento do residentes foram capazes de completar a tarefa no pós-teste.

**Conclusão** O modelo de treino usando a técnica da Regra do Gladiador para sutura laparoscópica melhora o tempo de atar nós com uma performance estatisticamente similar, não havendo diferenças quanto ao ano da residência, após treinamento sistematizado.

## Introduction

In recent years, the exponential growth in medical technologies has brought a range of new tools to the repertoire of minimally invasive gynecological surgery. Laparoscopic surgery has become an integral part of this practice, with benefits well documented in the literature. 1 2 Thus, it is necessary to create effective and reproducible training protocols for residents in laparoscopic surgery. The traditional Hallsteadian training model has been used by several academic centers during training in laparoscopic surgery. 3 4 Unfortunately, this method has been shown to be highly subjective and ineffective. 5 Some characteristics of laparoscopy are not suitable for this method, such as the slower learning curve to develop the hand-eye coordination necessary to operate by a video monitor, the interpretation of the limitations of the two-dimensional image, the reduction of tactile feedback and the fulcrum effect. 6 It should also be noticed that when a novice surgeon has to develop these skills in the operating room, the cost of surgery and operative time significantly increase. 7

Currently, most surgical educators understand that basic skills in laparoscopy should be taught initially outside the operating room; most surgical training programs use a variety of models, including inanimate models, virtual reality, training in live animals or in corpse. 8 Simulation-based training has been shown to improve the residents' laparoscopic skills and can also be used to assess their proficiency objectively. 9 10 Laparoscopic training based on proficiency was developed for training boxes (box training – BT), which proved to be an effective method to develop laparoscopic surgical skills. 11 12

The suture consists of processes of knotting and closing of a blocking sequence; this task was initially considered a major limiting factor in laparoscopic surgeries, but now it is considered a basic skill to perform these procedures. The wires used have several specific characteristics that directly influence the difficulty of the suture (mono or multifilament; absorption rate; elasticity; tensile strength; easy handling and duration of the inflammatory reaction). 13

The objective of the present study was to evaluate the amount of time taken to make surgical laparoscopic knots using 2 different types of wires (polyglactin multifilament and polypropylene monofilament) with the previous knowledge of medical residents in gynecology and obstetrics compared with the time taken after participating in 4 training sessions in systematic BTs using the Gladiator rule and to compare the individual performances and those among different groups of residents according to their years into the residency program (PGY-1/R1, PGY-2/R2 and PGY-3/R3).

## Methods

The study is a prospective cohort, in which all residents in gynecology and obstetrics from the residency program at Maternidade Escola Assis Chateaubriand (MEAC) were selected, totaling 32 residents, ten R1, twelve R2, and ten R3. This service currently has a structured service for minimally invasive gynecological surgery and a specialization service (R4) in gynecological endoscopy. The inclusion criteria were to be a MEAC resident doctor, to be available to carry out the evaluations and training, and to carry out the pre and posttraining. Ethical approval was obtained under the register 55770422.0.0000.5050, issued by the Ethical Committee from Maternidade Escola Assis Chateaubriand at Universidade Federal do Ceará.

The resident doctors were initially submitted to a pre-training that consisted of making two blocking sequences, which could be composed of five semi-knots. Participants should use a polyglactin, multifilament, absorbable and synthetic wire, 0 (COVIDIEN) for the first blocking sequence, and a polypropylene wire, monofilament and non-absorbable, 2–0 (SURGIPRO II - COVIDIEN) for the second sequence in a maximum time of 30 minutes (min) for both knots, only with their previous knowledge. Romeu and Minelli Gladiator rule was used to teach trainees how to tie intracorporeal knots. 14

Next, we made 4 laparoscopic suture training meetings, each one with an emphasis on a specific skill: 1st meeting - horizon, home base and Gladiator rule (above and below the wire); 2nd meeting – ambidextrous, semi-knot, and semi-key; 3rd meeting – blocking sequence H3H2, H2H1SSB; 4th meeting - blocking sequence. Simulation models of the abdominal cavity, support model for the realization of the knots, 1 laparoscopic needle holder and a Maryland forceps were used in the training. 14 After training, a second test was performed in the same pattern as the previous one, and the time to make the knot was again evaluated.

Due to changes in rounds and difficulties in attending training, our sample was reduced to 10 R3, 5 R2, and 5 R1. Data collection was performed during the months of November and December 2018 and January 2019.

Regarding the statistical analysis of the numerical variables, the data will be presented in mean and standard deviation, and in median. In the categorical variables, the data will be exposed in frequency and prevalence rate to investigate associations between the variables evaluated and the result of the pretraining test in comparison with the posttraining test and among the groups of residents (PGY-1, PGY-2, and PGY-3). In the analysis of the characteristics of the groups, the Kruskall-Wallis test and the paired Student *t* -test were used, conditioned to the adherence of the data to the Gaussian distribution. A significance level of 5% was adopted. Statistical analyses were performed using the Jamovi statistical software version: 0.9.5.12 and Microsoft Excel 2016 (Microsoft Corp., Redmond, WA, USA).

## Results

**Fig. 1:**
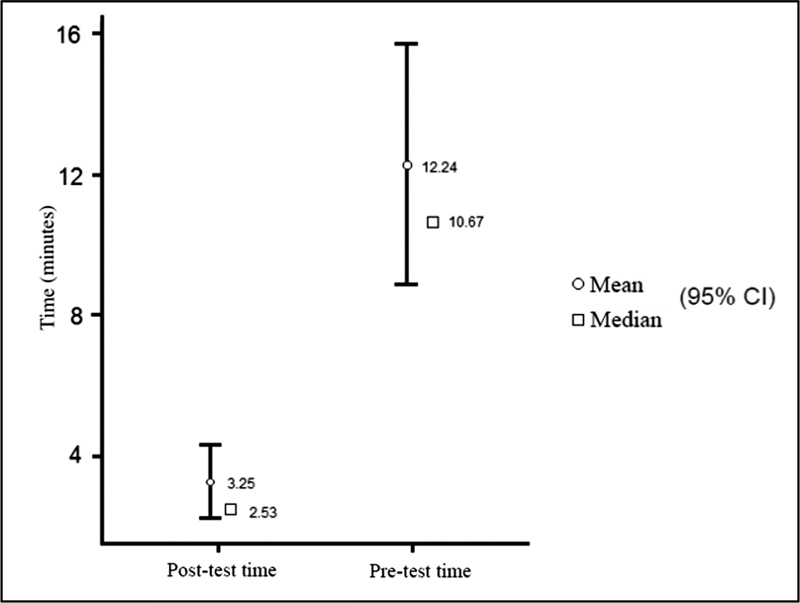
Pre vs Post training time using polyglactin wire.

The results obtained by comparing the pre and posttraining times for making a knot with polyglactin wire ( Fig. 1 ) showed a statistically significant improvement, with an initial median of 10.67 minutes and a mean of 12.24 minutes, which evolved to a median of 2.53 minutes and mean of 3.25 minutes ( Table 1 ).

**Table 1:** Pre and post training time using polyglactin wire

	n	Mean(min)	Median	SD	SS
Pre training time	23	12:24	10:67	8.36	1.743
Post training time	23	3:25	2:53	2.56	0.534

Abbreviations: SD, standard deviation; SS, statistical significance.

In regard to statistical analysis, these results are statistically significant, with *p* < 0.01 ( Table 2 ).

**Table 2:** Wilcoxon test – polyglactin wire

			Statistics	*p*
Post training time	Pre training time	Wilcoxon W	2.00	< 0.01

**Fig. 2:**
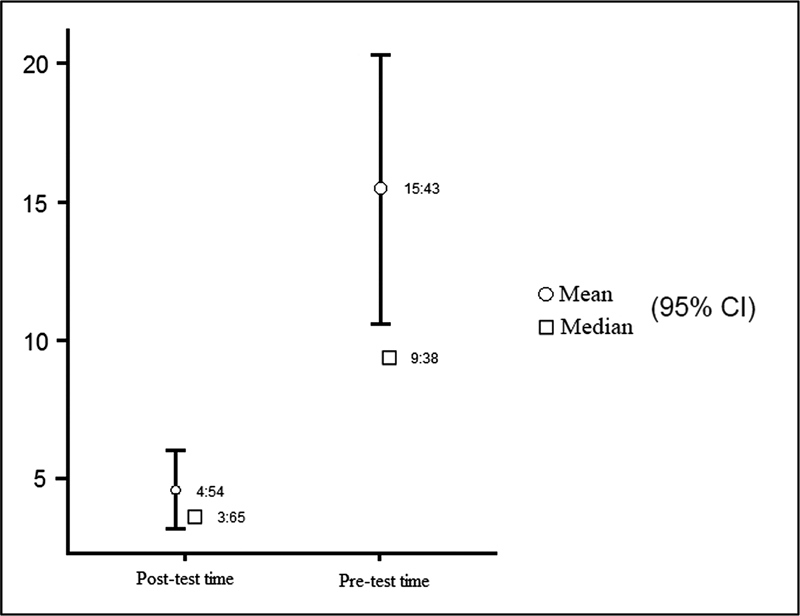
Pre vs Post training time using polypropylene wire.

The time evaluation using the polypropylene wire ( Fig. 2 ) presented a median of 9.38 minutes with a mean of 15.43 minutes in the pretraining, which evolved to a median of 3.65 minutes with a mean of 4.54 minutes posttraining ( Table 3 ).

**Table 3:** Pre and posttraining time using polypropylene wire

	n	Mean(min)	Median	SD	SS
Pre training time	23	15:43	9:38	11.89	2.480
Post training time	23	4:54	3:65	3.47	0.723

These results are also statistically significant, with *p* < 0.05 ( Table 4 ).

**Table 4:** Wilcoxon test – polypropylene wire

			Statistics	*p*
Post training time	Pretraining time	Wilcoxon W	16.00	< 0.01

During pretraining evaluations, 64.2% of the residents were able to make a knot with polypropylene wire. In contrast, 100% of the residents were able to complete the task posttraining. The proportion of residents who managed to make the knot before and after training was also evaluated. In pretraining evaluation, the result was not different from 50%, without statistical significance ( *p* = 0.210). In the posttraining, the result was different from 50%, with statistical significance ( *p* < 0.01). Therefore, there was a significant evolution in all groups after training. We also evaluated time improvement by comparing the groups in different years of residence (PGY-1, PGY-2, and PGY-3). The assessment showed improvement by reducing the time to perform uniform knots among the residency groups but did not show statistical evidence regarding skill enhancement.

## Discussion

Our study demonstrated a significant time improvement in making laparoscopic surgical knots performed by a resident doctor, after four training sessions in laparoscopic sutures. These performances' improvement results were observed using polyglactin or polypropylene wire. We observed at the beginning of the study, before the resident doctors underwent training, that only 64.2% were able to make the knot with polypropylene. The greater ease with multifilament sutures was very clear, limiting the residents' competence to make knots with monofilament sutures. In the literature, we find several references that describe different specific characteristics of the wires used in sutures that directly influence the difficulty of their manipulation (mono or multifilament; absorption rate; elasticity; tensile strength; easy handling and duration of the inflammatory reaction). 13 Thus, there is an urgent need for the resident physician to acquire competence and proficiency in performing laparoscopic sutures with different types of wires available.

The conformation of the knot is a key point, and its security is defined as a sequence of knots that does not untie or slip and opens before the wire breaks or does not slip by > 3 mm. 13 15 16 More recently, with dynamometric evaluation, the safe knot was redefined as the greatest pressure sustained by it until it slips, fails, or the suture breaks. 17 According to our data, systematic training with mentors in a simulated BT environment is able to increase the proficiency of resident physicians in a few sessions. We observed in the posttraining evaluations, performed after 4 training sessions in laparoscopic sutures, that 100% of the students were able to successfully perform a suture also with polypropylene wire.

The traditional Halstedian learning model—“seeing one, making one, teaching one”—has been challenged recently. The learning opportunity in the operating room has decreased, mainly because of time pressure, cost, and, above all, bioethical and medical-legal issues. 6 The best way to develop laparoscopic surgical skills and overcome this learning curve seems to be by participating in formal training in a specialized surgical skills laboratory. 5 6 Laparoscopic suture, for example, is an advanced laparoscopic skill that allows the surgeon to expand the application in various dimensions of video surgery. 8 However, it consists of a skill that is difficult to develop and requires many hours of specialized training. 9

Suturing and tying knots are essential skills in most surgical procedures for tissue apposition and hemostasis. 5 We recognize as fundamental the need for all surgeons to understand and master the correct combinations of surgical knots. 10 When a suture fails, the consequences can be disastrous, with possible massive bleeding, evisceration and dehiscence of the vaginal dome. 13 Many different training systems have been designed and manufactured to allow students and surgeons to acquire some skill in handling dedicated equipment and performing laparoscopic surgical procedures. These different training systems can be separated into physical simulation (box trainer or animal model) and systems that use virtual software. 11 12 18 19 20 21 22 23 24

In addition to an adequate environment and simulation materials for training in laparoscopic sutures, it is essential that we have standardization and methodization in teaching and learning technologies. In our study, all training was based on the Gladiator rule. In 2017, Romeo et al. studied 2,000 thousand knots made by experienced and trained surgeons using the Gladiator's suturing technique followed by tests with a dynamometer and concluded that the blocking sequences composed of 5 semi-knots or semi-keys had the best results withstanding pressures greater than 30 Newtons (safe pressure for gynecological procedures). 18

In our study, when evaluating groups of resident physicians from different years, we observed that there was no statistically significant difference in the improvement of suturing time. These findings are similar to those from Galvão-Neto et al., who demonstrated that previous medical experience is valid only when medical decision-making is necessary and does not seem to be relevant in the final result in a scenario of simulation of skills. 11 12 We conducted our training based on the Gladiator rule, a methodology already validated by Liceaga et al., in 2013, with good results in teaching. 19

The results of substantial time improvement to perform the suture found in our study corroborate with the findings by Assencio et al., who also used the Gladiator rule. However, they performed 14 hours of theoretical and practical training with polyglactin wire. In our study, unlike Assencio et al., each resident underwent only 1 hour and 20 minutes of practical training, and we also tested the knotting of polypropylene thread. According to our findings, we dare to infer that shorter, fractional training, focused on mentored and systematized practice would have a similar result in improving time in knotting. We demonstrated that, at the end of the training, both residents of the first year without experience in laparoscopy and those in more advanced periods reached proficiency without a statistically significant difference in number and in resistance of the stitches, creating less dispersion and more homogeneous results. We believe, therefore, that it is fundamentally and fully possible to learn the intracorporeal suture at the beginning of the endoscopic learning curve and not only in the case of an experienced and skilled surgeon. In fact, it must be considered that also during basic operative procedures, the surgeon may unexpectedly need the ability to intracorporeal suture. The technique taught during this type of course, as demonstrated in the present work, is essential to improve dexterity and coordination, especially among inexperienced laparoscopists.

Another observation of our study was the proportional time decrease to perform the suture with polypropylene when compared with polyglactin. Therefore, the hypothesis is refuted that it is impossible to improve the quality of the suture with different materials, with different physical and chemical characteristics for beginners. In this way, the possible use of different materials in different situations is made available to inexperienced surgeons, increasing their effectiveness and safety of laparoscopic sutures.

Although it was not the objective of the study's outcome, our results somehow corroborate with the literature regarding the effectiveness of BTs for the training of laparoscopic skills such as psychomotor control, spatial orientation and improvement of two-dimensional vision, basic concepts for the development of laparoscopic suture. 19 20 21 22 23

Despite the evidence on simulated training, its implementation is unfortunately still not systematic. Among the difficulties in implementing this training, we can mention the required time for training, the high cost of training material and the lack of access to the simulators. 24 25 Teaching surgical skills has undergone severe changes after the development of laparoscopy, when it was necessary to develop new teaching protocols to improve efficiency and safety in surgeries. Our study corroborated with the findings of other studies, emphasizing the need for this type of training protocol, and once again validating its use in in-service training. 26

## Conclusion

We find that the training for laparoscopic suture skills using the Gladiator rule improves the knotting time performed by trainees with both polyglactin and polypropylene wires. Furthermore, regardless of the year of resident physicians, the improvement was the same after methodical training.
